# Provider and patient perceptions of traditional Chinese medicine service pricing reform: evidence from Northeast China

**DOI:** 10.3389/fpubh.2026.1846076

**Published:** 2026-06-08

**Authors:** Fazheng Zhao, Yichi Chen, Ruoxin Wang, Xinyi Wu, Xin Tong

**Affiliations:** 1 Zhejiang Cultural Research Institute of Chinese Medicine, Zhejiang Chinese Medical University, Hangzhou, China; 2 College of Humanities and Management, Zhejiang Chinese Medical University, Hangzhou, China; 3 School of Basic Medicine, Heilongjiang University of Chinese Medicine, Harbin, China

**Keywords:** traditional Chinese medicine services, healthcare service prices, provider perception, willingness to pay, health equity

## Abstract

**Background:**

In 2025, China issued the Guidelines for the Establishment of Pricing Items for TCM Medical Services, aimed at shifting TCM services toward value-based pricing. This study examined provider, patient, and administrator perceptions of TCM pricing reform in Northeast China.

**Methods:**

A survey was conducted in Jilin and Liaoning provinces, yielding 399 valid questionnaires, including 250 from healthcare professionals, 125 from patients, and 24 from healthcare security and medical administration staffs. Healthcare professional data were analyzed using multivariate regression, patient data described demand-side perceptions and stated pricing preferences, administrator data provided contextual evidence.

**Results:**

Among healthcare professionals, professional title (*β* = −0.129, *p* = 0.017) and medical insurance payment restrictions (*β* = −0.236, *p* = 0.000) were negatively associated with pricing and payment satisfaction, while hospital management support was positively associated with satisfaction (*β* = 0.396, *p* = 0.000). Implementation-barrier models had low explanatory power and were interpreted as exploratory. Patients showed limited direct awareness of pricing item or billing changes, but reported tension between affordability and service value: 54.0% favored moderately lower prices, 39.5% supported moderate price increases with better service quality, and 37.9% supported tiered pricing. Logistic analyses identified no clear respondent level predictors. Administrators highlighted cost-accounting complexity and weak coordination between pricing reform and insurance payment.

**Conclusion:**

TCM service pricing reform is a health system coordination issue involving provider incentives, patient affordability, insurance payment, and equitable access. The findings support value-based cost accounting, tiered pricing and access mechanisms, improved patients value communication, and closer pricing–insurance coordination.

## 1 Introduction

The deepening of healthcare system reform has entered a new phase characterized by “system integration and coordinated advancement”, in which deepening the reform of healthcare service pricing is a critical component of building a high-quality healthcare system. The integration of traditional, complementary, and integrative medicine into health systems has become an important issue in global health policy. The World Health Organization’s Global Traditional Medicine Strategy 2025–2034 emphasizes evidence-based integration, safety, people-centered care, and universal access to traditional, complementary, and integrative medicine (1). From the perspective of universal health coverage and value-based service delivery, the pricing of TCM services is not merely a technical matter of fee schedules (2). It affects provider incentives, patient affordability, insurance payment arrangements, and the equitable distribution of healthcare resources. TCM diagnosis and treatment are often experience-based and individualized, and the value of many TCM techniques is difficult to quantify through equipment or material consumption alone. For this reason, the pricing system has long faced challenges such as inadequate recognition of labor value and imprecise classification of service items (3).

Research on healthcare pricing and payment suggests that payment mechanisms can shape provider behavior, service delivery incentives, efficiency and accessibility (4). Value-based healthcare frameworks further argue that payment should reflect outcomes, quality, and the resources required to deliver care rather than relying only on itemized inputs or material costs (5, 6). At the same time, diagnosis-related group and other prospective payment systems show that reimbursement rules may improve transparency and cost control, but they may also create tensions when payment categories do not adequately reflect the clinical logic of particular services (7, 8). These issues are especially relevant for TCM services, whose value is often embedded in individualized diagnosis relevant for TCM services, whose value is often embedded in individualized diagnosis, syndrome differentiation, manual techniques, time input, and practitioner experience.

In 2025, China issued the guidelines for the establishment of pricing items for TCM medical services, providing a policy setting for examining these issues (9, 10). The guidelines cover TCM therapies (moxibustion, cupping, and tuina), external TCM treatments, acupuncture, orthopedics, and special TCM therapies. They aim to promote service quality and efficiency by standardizing pricing items, thereby facilitating a transition toward a value-based approach that prioritizes technical expertise and professional services. However, implementation may vary across regions and institutions because pricing reform interacts with local service capacity, insurance payment rules, cost accounting practices, and patient affordability.

Existing studies on TCM pricing reform in China have mainly focused on pricing item design, hospital-level implementation, cost accounting, or regional price comparison (3, 10–12). Fewer empirical studies have simultaneously examined provider perceptions, patient-side stated pricing preferences, and administrative implementation constraints. However, it is precisely this type of research that is of great significance for promoting the transition of TCM services toward a value-based model centered on technical expertise and professional services. The Northeast region of China provides a valuable empirical context for this purpose. The provinces of Jilin and Liaoning possess a solid foundation in TCM services while also exhibiting variations among medical institutions at all levels in terms of resources, management, service capacity, and policy implementation capabilities, which facilitates the testing and advancement of relevant research.

This study therefore had four objectives. First, it aimed to describe healthcare professional, patient, and administrator perceptions of TCM pricing reform in Jilin and Liaoning provinces. Second, it sought to identify provider-side factors associated with pricing and payment satisfaction and perceived implementation barriers. Third, it examined patient-side perceptions of price–quality alignment and stated pricing preferences. Fourth, it discussed the implications of these findings for value-based pricing, insurance payment coordination, and equitable access to TCM services.

## 2 Methods

### 2.1 Survey participants

This study adopted a multi-stage stratified sampling strategy. First, Jilin and Liaoning provinces were selected as representative provinces in Northeast China because they have a strong TCM service foundation and varying levels of economic and healthcare resource development. Second, Shenyang, Dalian, Changchun, and Dandong were selected to cover provincial capitals, economically developed cities, and cities with different levels of TCM service supply. Third, medical institutions at different levels were included, covering provincial TCM hospitals or integrated traditional Chinese and Western medicine hospitals, TCM departments in general hospitals, municipal or county-level TCM hospitals, community health service centers, township health centers, and privately-run TCM clinics. The survey participants consisted of three groups: healthcare professionals providing TCM services, patients who had used TCM services, and staffs from health commissions, TCM administration departments, or medical insurance bureaus. Healthcare professionals were eligible if they had at least 1 year of clinical experience related to TCM services. Patients were eligible if they were aged 18 years or above and had used TCM medical services within the previous 12 months. Administrator questionnaires were collected from staffs familiar with TCM service pricing, medical insurance payment, or policy implementation.

The sample size was assessed separately according to the analytical role of each sub-sample. The healthcare professional sample was the primary sample for multivariate regression analysis. With 250 valid responses, it met the commonly used rule of at least 10 observations per predictor variable for multiple regression models. The patient sample included 125 valid responses and was used mainly for descriptive analysis of demand-side perceptions and willingness to pay. The administrator sample included 24 valid responses and was not used for regression modeling, instead, it provided contextual evidence on policy implementation and administrative perspectives. The item-to-response ratio was used only as a supplementary reference for questionnaire coverage, rather than as the sole basis for determining the adequacy of OLS regression sample size.

### 2.2 Survey instrument and data collection

The questionnaires were developed in Chinese because all respondents were recruited in China. Questionnaire development followed a policy implementation logic rather than the direct adoption of a validated psychometric scale. First, the research team reviewed the 2025 Guidelines for the Establishment of Pricing Items for TCM Medical Services, relevant provincial dynamic price adjustment policies, and prior literature on healthcare pricing, provider payment, and patient willingness to pay. Second, separate questionnaires were designed for healthcare professionals, patients, and administrators according to their roles in TCM service provision, utilization, and policy implementation. Third, the items were discussed and revised by project-team members to improve content relevance, clarity, and consistency with local implementation practice. No formal pilot testing was conducted, and no external expert review was undertaken.

The healthcare professional questionnaire covered demographic characteristics, professional background, institutional type, policy familiarity, perceived implementation barriers, hospital management support, and satisfaction with pricing and payment. The patient questionnaire covered sociodemographic characteristics, TCM service utilization, awareness of pricing changes, perceived service quality, perceived price-effectiveness alignment, and willingness to pay. The administrator questionnaire focused on policy implementation stage, supporting measures, interdepartmental coordination, implementation difficulties, and suggested policy improvements. English descriptions of questionnaire items and variables in the manuscript were translated by the research team and checked for conceptual equivalence. Data were collected through a combination of on-site recruitment and electronic questionnaires. Investigators distributed questionnaire links or QR codes in outpatient areas, physician offices, and relevant administrative settings. All questionnaires were anonymous. Responses with incomplete key information, obvious logical contradictions, or extremely short completion time were excluded.

### 2.3 Variable measurement and statistical methods

Descriptive statistics were used to summarize participant characteristics and attitudes toward TCM service pricing reform. Counts and percentages were reported for categorical variables. The key dependent variables in the healthcare professional models were single five-point Likert-type items, including pricing and payment satisfaction and perceived implementation barrier scores. The disconnect score referred to the single item measuring the perceived discrepancy between project guidelines and clinical practice, scored from 1 to 5, with higher scores indicating stronger perceived barriers. Policy familiarity was calculated as the mean score of three items measuring familiarity with relevant TCM laws, national pricing item guidelines, and provincial dynamic price adjustment policies.

Cronbach’s α was not calculated for the main dependent variables because they were measured using single policy-perception items rather than multi-item latent scales. For multi-item indices used as explanatory or contextual measures, internal consistency was assessed where appropriate. The three-item policy-familiarity index showed high internal consistency (Cronbach’s α = 0.907), and the four-item implementation barrier set showed good internal consistency (Cronbach’s α = 0.883).

The selection of regression variables was guided by an analytical framework linking healthcare service price perception with individual characteristics, occupational seniority, institutional context, and policy implementation conditions. Core explanatory variables included medical insurance payment restrictions, hospital management support, guideline coverage, training frequency, institutional level, policy familiarity, and coverage of ethnic medical techniques. To reduce omitted-variable bias, gender, age, working mechanism, and professional title were included as demographic and occupational covariates. The exact number of years of service was not collected in the healthcare professional questionnaire; therefore, age and professional title were used as available indicators of career stage, this limitation is acknowledged in the limitation.

Multiple linear regression was retained as the main model because the five-point outcomes were treated as approximately continuous policy-perception scores and because OLS coefficients are straightforward to interpret as average score differences. However, because the outcomes were ordered categorical responses, ordered logit and ordered probit models using the same predictor sets were estimated as robustness checks. For OLS models, *β* coefficients, standard errors, 95% confidence intervals, *p* values, variance inflation factors, *R*^2^, adjusted *R*^2^, *F* statistics, and model *p* values were reported. Multicollinearity was assessed using VIF values. Breusch-Pagan tests and HC3 robust standard errors were used as sensitivity checks for heteroscedasticity. Given the cross-sectional design and the examination of several related outcomes, the regression results were interpreted as exploratory associations rather than confirmatory causal estimates.

For the patient sample, descriptive statistics were used as the primary analysis because the sample size was modest and the patient questionnaire was designed mainly to capture demand-side perceptions. To respond to the centrality of stated willingness to pay, two exploratory binary logistic regression models were additionally estimated. The dependent variables were whether the patient selected moderate price increases with improved service quality and whether the patient selected tiered pricing. Predictors included age group, TCM use frequency, perceived price burden, out-of-pocket share, efficacy-price alignment, and support for pay-for-effect. Odds ratios, 95% confidence intervals, *p* values, McFadden pseudo *R*^2^, and AIC were reported. These patient-side models were interpreted as exploratory analyses of stated pricing preferences rather than estimates of actual monetary willingness to pay. Administrator sample were analyzed descriptively as contextual evidence for policy implementation. Data processing and statistical analysis were performed using SPSS 27.0 and Python 3.12.

## 3 Results

### 3.1 Analysis of perceived discrepancies in the value of medical services among healthcare workers in the northeast region (Jilin and Liaoning provinces)

#### 3.1.1 Socio-demographic characteristics of healthcare professionals participating in the study in the northeast region (Jilin and Liaoning provinces)

A total of 250 healthcare workers participated in the survey; see Table 1 for details.

**Table 1:** Sociodemographic characteristics of participating medical staff.

Variable	Category	Frequency	Percentage (%)
Gender	Male	90	36.0
	Female	160	64.0
Age	Ages 20–30	35	14.0
	Ages 31–40	68	27.2
	Ages 41–50	77	30.8
	Ages 51–60	60	24.0
	Ages 61 and older	10	4.0
Professional title	Entry-level (medical assistant/physician)	99	39.6
	Intermediate level (attending physician)	89	35.6
	Associate senior professional title	38	15.2
	Senior professional title	24	9.6
Working mechanism	Provincial traditional Chinese medicine hospital/hospital of integrated traditional and Western medicine	25	10.0
	Department of traditional Chinese medicine at a general hospital	24	9.6
	Municipal/county-level traditional Chinese medicine hospitals/ethnic medicine hospitals	92	36.8
	Community health service center/township health center	97	38.8
	Privately run traditional Chinese medicine clinics	12	4.8

#### 3.1.2 Factors influencing satisfaction with pricing and payment for TCM services

Regarding satisfaction with the current pricing and reimbursement of TCM service items in Jilin and Liaoning provinces, 54.4% of the surveyed healthcare professionals selected “somewhat satisfied,” and a cumulative 78.8% selected “somewhat satisfied” or higher. Those who selected “very dissatisfied” and “dissatisfied” combined accounted for 21.6%; see Table 2 for details. The values for the remaining variables are shown in Table 3.

**Table 2:** Satisfaction with pricing and payment of TCM medical service items in Jilin and Liaoning provinces.

Variable	Category	Frequency	Percentage (%)
Satisfaction with pricing and payment	Very dissatisfied	16	6.4
	Dissatisfied	38	15.2
	Generally satisfied	136	54.4
	Satisfied	45	18.0
	Very satisfied	15	6.0

**Table 3:** Variable assignment table.

Variable	Assignment
Gender	1 = male, 2 = female
Age	1 = ages 20–30, 2 = ages 31–40, 3 = ages 41–50, 4 = ages 51–60, 5 = ages 61 and older
Working mechanism	1 = Provincial TCM hospital/integrated TCM-Western medicine hospital, 2 = department of TCM at a general hospital, 3 = municipal/county-level TCM hospital/ethnic medicine hospital, 4 = community health service center/township health center, 5 = privately run TCM clinic
Professional title	1 = entry-level, 2 = intermediate level, 3 = associate senior professional title, 4 = senior professional title
Health insurance coverage limits	1 = 1 point, 2 = 2 points, 3 = 3 points, 4 = 4 points, 5 = 5 points
Satisfaction with the level of support from hospital management	1 = very dissatisfied, 2 = dissatisfied, 3 = generally satisfied, 4 = satisfied, 5 = very satisfied
Whether the guideline covers existing medical services	1 = yes, 2 = no

Multivariate linear regression analysis was conducted to identify factors associated with pricing and payment satisfaction for TCM services. After controlling for gender, age, working mechanism, and professional title, the model was statistically significant (*F* = 22.082, *p* = 0.000; *R*^2^ = 0.390; adjusted *R*^2^ = 0.372). Medical insurance payment restrictions were negatively associated with pricing and payment satisfaction (*β* = −0.236, SE = 0.037, 95% CI: −0.309 to −0.163, *p* = 0.000), while hospital management support was positively associated with satisfaction (*β* = 0.396, SE = 0.054, 95% CI: 0.291 to 0.502, *p* = 0.000). Professional title remained negatively associated with satisfaction after adjustment (*β* = −0.129, SE = 0.054, 95% CI: −0.235 to −0.024, *p* = 0.017). Whether the guidelines covered existing medical services was also negatively associated with satisfaction (*β* = −0.497, SE = 0.126, 95% CI: −0.744 to −0.249, *p* = 0.000). Gender, age, and working mechanism were not statistically significant. The Breusch-Pagan test suggested heteroscedasticity in this model; therefore, HC3 robust standard errors were examined as a sensitivity check, and the main significant associations remained unchanged. Detailed results are shown in Table 4.

**Table 4:** Multiple linear regression analysis of factors influencing satisfaction with pricing and payment of TCM medical service items.

Variable	*β*	SE	*t*	*p*	95% CI	VIF
Gender	0.161	0.098	1.638	0.103	−0.033 to 0.355	1.068
Age	−0.046	0.049	−0.951	0.343	−0.142 to 0.050	1.335
Working mechanism	−0.013	0.046	−0.291	0.771	−0.103 to 0.076	1.036
Professional title	−0.129	0.054	−2.410	0.017	−0.235 to −0.024	1.282
Health insurance coverage limits	−0.236	0.037	−6.344	0.000	−0.309 to −0.163	1.021
Satisfaction with the level of support from hospital management	0.396	0.054	7.401	0.000	0.291 to 0.502	1.096
Whether the guideline covers existing medical services	−0.497	0.126	−3.951	0.000	−0.744 to −0.249	1.098
Constant	2.996	0.400	7.496	0.000	2.209 to 3.784	-

The dependent variable is pricing and payment satisfaction; *N* = 250. The model includes gender, age, working mechanism, and professional title as demographic and occupational covariates; *R*^2^ = 0.390, adjusted *R*^2^ = 0.372; *F* = 22.082, *p* = 0.000. The maximum VIF was 1.335. HC3 robust standard errors were examined as a sensitivity check because the Breusch–Pagan test suggested heteroscedasticity (LM = 21.142, *p* = 0.004; *F* = 3.194, *p* = 0.003); the main significant associations remained unchanged.

#### 3.1.3 Factors influencing the scoring of implementation barriers for the guidelines on the approval of pricing items for TCM services

Healthcare professionals assigned an average score of 2.22 ± 1.099 (out of 5; same applies below) to the obstacle “disconnect between the project guidelines and clinical practice” in the implementation of the project guidelines for TCM medical service pricing items in Jilin and Liaoning provinces; they assigned an average score of 2.17 ± 1.048 to the obstacle “lack of a collaboration mechanism between TCM and Western medicine”; The mean score for the implementation obstacle “failure to include ethnic medical techniques in the guidelines” was 2.12 ± 1.079. The values for the remaining variables are shown in Table 5.

**Table 5:** Variable assignment table.

Variable	Assignment
Gender	1 = male, 2 = female
Age	1 = ages 20–30, 2 = ages 31–40, 3 = ages 41–50, 4 = ages 51–60, 5 = ages 61 and older
Working mechanism	1 = Provincial TCM hospital/integrated TCM-Western medicine hospital, 2 = department of TCM at a general hospital, 3 = municipal/county-level TCM hospital/ethnic medicine hospital, 4 = community health service center/township health center, 5 = privately run TCM clinic
Professional title	1 = Entry level, 2 = intermediate level, 3 = associate senior professional title, 4 = senior professional title
Organizational level	1 = 3A hospital, 2 = 3B hospital, 3 = 2A hospital, 4 = 2B hospital, 5 = community/township hospitals, 6 = private practice, 7 = ethnic medicine hospital
Whether in an ethnic autonomous region	1 = yes, 2 = no
Frequency of project proposal guidelines training	1 = at least once a month, 2 = once a quarter, 3 = once or twice a year, 4 = never implemented
Current guidelines cover traditional medical techniques	1 = full coverage, 2 = partial coverage, 3 = not covered at all
Mean score for policy familiarity	Mean of three policy familiarity items; higher scores indicate greater familiarity
Satisfaction with the level of support from hospital management	1 = very dissatisfied, 2 = dissatisfied, 3 = generally satisfied, 4 = satisfied, 5 = very satisfied

Multivariate linear regression analysis was used to examine factors associated with the score for the implementation barrier “discrepancy between project guidelines and clinical practice”. After controlling for gender, age, working mechanism, and professional title, the overall model approached conventional statistical significance but had limited explanatory power (*F* = 1.928, *p* = 0.057; *R*^2^ = 0.063; adjusted *R*^2^ = 0.030). Institutional level showed a marginal negative association with the disconnect score (*β* = −0.120, SE = 0.064, 95% CI: −0.247 to 0.007, *p* = 0.064), while the coverage of local ethnic medical techniques in current guidelines was positively associated with the disconnect score (*β* = 0.298, SE = 0.127, 95% CI: 0.048 to 0.547, *p* = 0.020). Other variables were not statistically significant. Detailed results are shown in Table 6.

**Table 6:** Multiple linear regression analysis of factors influencing the disconnect score between guideline establishment and clinical practice.

Variable	*β*	SE	*t*	*p*	95% CI	VIF
Gender	−0.097	0.150	−0.643	0.521	−0.393 to 0.199	1.084
Age	−0.067	0.075	−0.893	0.373	−0.216 to 0.081	1.384
Working mechanism	0.070	0.093	0.747	0.456	−0.114 to 0.253	1.830
Professional title	0.077	0.083	0.923	0.357	−0.087 to 0.241	1.350
Organizational level	−0.120	0.064	−1.862	0.064	−0.247 to 0.007	1.919
Frequency of project proposal guidelines training	−0.052	0.075	−0.696	0.487	−0.201 to 0.096	1.055
Current guidelines cover traditional medical techniques	0.298	0.127	2.350	0.020	0.048 to 0.547	1.103
Satisfaction with the level of support from hospital management	−0.131	0.082	−1.608	0.109	−0.292 to 0.030	1.113
Constant	2.612	0.592	4.410	0.000	1.445 to 3.779	-

The dependent variable is the score for the implementation barrier “discrepancy between project guidelines and clinical practice”; *n* = 240 after listwise deletion. The model includes gender, age, working mechanism, and professional title as demographic and occupational covariates; *R*^2^ = 0.063, adjusted *R*^2^ = 0.030; *F* = 1.928, *p* = 0.057. The maximum VIF was 1.919.

Multivariate linear regression analysis was conducted to examine factors associated with the score for the implementation obstacle “Lack of a collaboration mechanism between Traditional Chinese Medicine and Western medicine”. After controlling for gender, age, working mechanism, and professional title, the model was statistically significant but had low explanatory power (*F* = 2.401, *p* = 0.029; *R*^2^ = 0.058; adjusted *R*^2^ = 0.034). The coverage of local ethnic medical techniques in current guidelines was positively associated with the perceived lack of a collaborative mechanism (*β* = 0.284, SE = 0.122, 95% CI: 0.044 to 0.524, *p* = 0.021). Professional title showed a marginal positive association (*β* = 0.140, SE = 0.079, 95% CI: −0.015 to 0.295, *p* = 0.076), while gender, age, working mechanism, and hospital management support were not statistically significant. Detailed results are shown in Table 7.

**Table 7:** Multiple linear regression analysis of factors influencing the absence of integrated traditional Chinese medicine and Western medicine collaborative mechanism score.

Variable	*β*	SE	*t*	*p*	95% CI	VIF
Gender	−0.155	0.146	−1.062	0.290	−0.441 to 0.132	1.084
Age	−0.077	0.072	−1.065	0.288	−0.219 to 0.065	1.348
Working mechanism	−0.013	0.067	−0.186	0.852	−0.145 to 0.120	1.017
Professional title	0.140	0.079	1.785	0.076	−0.015 to 0.295	1.279
Current guidelines cover traditional medical techniques	0.284	0.122	2.333	0.021	0.044 to 0.524	1.083
Satisfaction with the level of support from hospital management	−0.126	0.078	−1.606	0.110	−0.280 to 0.029	1.095
Constant	2.295	0.554	4.144	0.000	1.204 to 3.386	-

The dependent variable is the score for the implementation barrier “lack of a collaboration mechanism between traditional Chinese medicine and Western medicine” in the project guidelines; *n* = 240 after listwise deletion. The model includes gender, age, working mechanism, and professional title as demographic and occupational covariates; *R*^2^ = 0.058, adjusted *R*^2^ = 0.034; *F* = 2.401, *p* = 0.029. The maximum VIF was 1.348.

Multivariate linear regression analysis was conducted to identify factors associated with the score for the implementation barrier “traditional medical techniques not included in the project guidelines”. After controlling for gender, age, working mechanism, and professional title, the model was statistically significant but still explained only a small proportion of variance (*F* = 3.691, *p* = 0.000; *R*^2^ = 0.113; adjusted *R*^2^ = 0.083). Institutional level was negatively associated with this barrier score (*β* = −0.234, SE = 0.062, 95% CI: −0.356 to −0.111, *p* = 0.000), while the coverage of ethnic medical techniques in current guidelines was positively associated with the score (*β* = 0.257, SE = 0.118, 95% CI: 0.024 to 0.490, *p* = 0.031). Gender was also associated with the barrier score (*β* = −0.326, SE = 0.145, 95% CI: −0.613 to −0.040, *p* = 0.026). Mean policy familiarity score was negatively associated with the barrier score (*β* = −0.160, SE = 0.067, 95% CI: −0.293 to −0.027, *p* = 0.018). Age, working mechanism, professional title, whether the institution was located in an ethnic autonomous region were not statistically significant. Detailed results are shown in Table 8.

**Table 8:** Multiple linear regression analysis of factors influencing the failure to incorporate ethnic medicine techniques into guideline establishment score.

Variable	*β*	SE	*t*	*p*	95% CI	VIF
Gender	−0.326	0.145	−2.245	0.026	−0.613 to −0.040	1.090
Age	−0.091	0.073	−1.244	0.215	−0.235 to 0.053	1.398
Working mechanism	0.145	0.090	1.610	0.109	−0.032 to 0.323	1.378
Professional title	0.032	0.081	0.398	0.691	−0.128 to 0.192	1.842
Organizational level	−0.234	0.062	−3.763	0.000	−0.356 to −0.111	1.919
Whether in an ethnic autonomous region	−0.134	0.323	−0.414	0.679	−0.769 to 0.502	1.020
Current guidelines cover traditional medical techniques	0.257	0.118	2.173	0.031	0.024 to 0.490	1.032
Mean score for policy familiarity	−0.160	0.067	−2.378	0.018	−0.293 to −0.027	1.078
Constant	3.459	0.827	4.183	0.000	1.830 to 5.089	-

The dependent variable is the score for the barrier to implementing the project guidelines, specifically “traditional ethnic medical techniques not included in the project guidelines”; *n* = 240 after listwise deletion. The model includes gender, age, working mechanism, and professional title as demographic and occupational covariates; *R*^2^ = 0.113, adjusted *R*^2^ = 0.083; *F* = 3.691, *p* = 0.000. The maximum VIF was 1.919.

Because the dependent variables were five-point ordered responses, ordered logit and ordered probit models were additionally estimated using the same covariate sets. The robustness checks generally supported the main OLS findings. In the pricing satisfaction model, professional title, health insurance coverage limits, hospital management support, and guideline coverage remained statistically significant with unchanged directions in both ordered logit and ordered probit models. For implementation barriers, coverage of traditional medical techniques remained positively associated with the guideline-clinical disconnect and TCM-Western collaboration-mechanism scores. For the ethnic technique inclusion barrier, gender, organizational level, coverage of traditional medical techniques, and mean policy familiarity score remained significant with consistent directions in both ordered logit and ordered probit models. As a sensitivity check for multiple testing across OLS predictor *p* values, the Benjamini-Hochberg FDR procedure indicated that health insurance coverage limits, hospital management support, guideline coverage, and organizational level remained the most robust associations after FDR adjustment. Given the low adjusted *R*^2^ values in the implementation barrier models, these findings should be interpreted as exploratory associations rather than strong predictive models. Detailed results are summarized in Table 9.

**Table 9:** Ordered logit and ordered probit robustness checks for key OLS findings.

Model	Key variable	OLS β(*p*)	Ordered logit β(*p*)	Ordered probit β(*p*)	Direction
Pricing satisfaction	Professional title	−0.129 (0.017)	−0.410 (0.007)	−0.193 (0.021)	Consistent
Pricing satisfaction	Health insurance coverage limits	−0.236 (0.000)	−0.691 (0.000)	−0.367 (0.000)	Consistent
Pricing satisfaction	Satisfaction with the level of support from hospital management	0.396 (0.000)	1.236 (0.000)	0.616 (0.000)	Consistent
Pricing satisfaction	Whether the guideline covers existing medical services	−0.497 (0.000)	−1.351 (0.000)	−0.744 (0.000)	Consistent
Guideline–clinical disconnect	Current guidelines cover traditional medical techniques	0.298 (0.020)	0.581 (0.008)	0.322 (0.012)	Consistent
TCM–Western collaboration barrier	Current guidelines cover traditional medical techniques	0.284 (0.021)	0.595 (0.006)	0.339 (0.008)	Consistent
Ethnic technique inclusion barrier	Gender	−0.326 (0.026)	−0.519 (0.046)	−0.314 (0.041)	Consistent
Ethnic technique inclusion barrier	Organizational level	−0.234 (0.000)	−0.394 (0.000)	−0.229 (0.000)	Consistent
Ethnic technique inclusion barrier	Current guidelines cover traditional medical techniques	0.257 (0.031)	0.479 (0.026)	0.285 (0.025)	Consistent
Ethnic technique inclusion barrier	Mean score for policy familiarity	−0.160 (0.018)	−0.357 (0.004)	−0.199 (0.006)	Consistent

Ordered logit and ordered probit models used the same predictor sets as the corresponding OLS models. OLS tables report β , SE, *t*, *p*, and VIF.

### 3.2 Analysis of patient perceptions in the northeast region (Jilin and Liaoning provinces)

#### 3.2.1 Characteristics of patients participating in the study in the northeast region (Jilin and Liaoning provinces)

A total of 125 patients participated in the questionnaire survey; see Table 10 for details.

**Table 10:** Sociodemographic characteristics of participating patients.

Variable	Category	Frequency	Percentage (%)
Age	Ages 18–30	78	62.4
	Ages 31–45	23	18.4
	Ages 46–60	12	9.6
	Ages 61 and older	12	9.6
Occupation	Regular employees	34	27.2
	Civil servants/public institution employees	22	17.6
	Self-employment	7	5.6
	Retirement	15	12.0
	Other occupations	47	37.6
Number of times receiving traditional Chinese medicine treatment within a year	Never	25	20.0
	1–3 times	80	64.0
	4–6 times	9	7.2
	7 times or more	11	8.8

#### 3.2.2 Patients’ perceptions of changes in pricing for TCM services

Data in Table 11 show that only 0.8% of patients reported noticing changes in TCM service names or billing methods, indicating that the technical details of pricing reform were largely invisible to patients. Therefore, the subsequent patient-side results should be interpreted as perceived changes in recent TCM service experience rather than direct awareness of the pricing reform itself. Regarding recent service quality experience, 49.6% of patients believed there was “no change,” while a combined 45.6% believed there was “a slight improvement or better”. In addition, 17.6% of patients explicitly reported service reduction such as shorter treatment times, while 42.4% selected “did not notice”. Regarding the alignment between the efficacy of TCM services and their prices, 66.4% of patients believed that efficacy and price were “fully aligned” or “fairly aligned.”

**Table 11:** Patients’ perceptions of price changes in TCM medical service items.

Dimensions of perception	Item	Frequency	Percentage (%)
Noticed changes in TCM item names or pricing	Yes	1	0.8
	No	124	99.2
Service quality change after TCM price adjustment	Markedly improved	24	19.2
	Slightly improved	33	26.4
	No change	62	49.6
	Slightly declined	3	2.4
	Markedly declined	3	2.4
Experienced service reduction due to price adjustment	Yes	22	17.6
	No	50	40.0
	Not noticed	53	42.4
TCM efficacy–price alignment	Fully matched	28	22.4
	Partially matched	55	44.0
	Moderately matched	34	27.2
	Somewhat mismatched	7	5.6
	Completely mismatched	1	0.8

Regarding recommendations for adjusting the prices of TCM services, data in Table 12 show that more than half (54.0%) of patients chose “moderately lower,” highlighting their perception of financial strain caused by current prices. 39.5% of patients chose “moderately higher, but with improved service quality”, indicating stated openness to quality-linked price increases. 37.9% of patients selected “tiered pricing”, reflecting support for differentiating TCM service fees by service quality, provider expertise, or institutional level. Only 26.6% of patients chose “maintain the status quo”. These findings should be interpreted as stated pricing preferences rather than estimates of actual monetary willingness to pay.

**Table 12:** Multiple response analysis of suggestions for TCM price adjustment.

Recommendations on TCM pricing	Response		
	Number of cases	Percentage (%)	Percentage of cases (%)
Keep current	33	16.8	26.6
Moderately decrease	67	34.2	54.0
Moderately increase with improved service quality	49	25.0	39.5
Differential pricing	47	24.0	37.9
Total	196	100.0	158.1

To further examine patient-side stated pricing preferences, exploratory binary logistic regression models were estimated. The first model used selection of “moderate price increases with improved service quality” as the dependent variable, and the second model used support for tiered pricing as the dependent variable. Predictors included age group, TCM use frequency, perceived price burden, out-of-pocket share, perceived efficacy-price alignment, and support for pay-for-effect. In both models, none of the predictors reached statistical significance, indicating that the available sociodemographic and perception variables did not clearly explain patient stated pricing preferences. Detailed results are shown in Tables 13, 14.

**Table 13:** Logistic regression analysis of quality-linked price-increase preference.

Variable	*B*	SE	*p*	OR	95% CI for OR
Age group	0.094	0.198	0.635	1.098	0.746–1.617
TCM use frequency	−0.047	0.244	0.849	0.955	0.592–1.540
Perceived price burden	0.063	0.200	0.753	1.065	0.719–1.577
Out-of-pocket share	−0.067	0.182	0.712	0.935	0.654–1.337
Efficacy–price alignment	0.290	0.283	0.305	1.337	0.767–2.330
Support for pay for effect	−0.035	0.224	0.877	0.966	0.622–1.499
Constant	−1.47	1.741	0.399	0.23	

The model used selection of “moderate price increases with improved service quality” as the dependent variable (*n* = 125; event *n* = 49; McFadden pseudo *R*^2^ = 0.010; AIC = 179.810). This model is exploratory because the patient sample was modest and the questionnaire measured stated pricing preferences rather than monetary willingness to pay.

**Table 14:** Logistic regression analysis of tiered pricing preference.

Variable	*B*	SE	*p*	OR	95% CI for OR
Age group	−0.138	0.204	0.499	0.871	0.584–1.299
TCM use frequency	−0.017	0.245	0.944	0.983	0.608–1.589
Perceived price burden	0.062	0.202	0.758	1.064	0.717–1.579
Out-of-pocket share	0.032	0.183	0.859	1.033	0.721–1.479
Efficacy–price alignment	−0.301	0.278	0.280	0.740	0.429–1.278
Support for pay-for-effect	0.053	0.224	0.814	1.054	0.679–1.637
Constant	0.458	1.725	0.791	1.580	

The model used selection of “tiered pricing” as the dependent variable (*n* = 125; event *n* = 47; McFadden pseudo *R*^2^ = 0.014; AIC = 177.123). This model is exploratory because the patient sample was modest and the questionnaire measured stated pricing preferences rather than monetary willingness to pay.

### 3.3 Administrative perspectives on policy implementation

The 24 administrator questionnaires were used as contextual evidence rather than for regression modeling. Among administrators, the most frequently reported implementation difficulties were complex cost accounting for TCM services (70.8%), conflicts in coordination with medical insurance payment policies (58.3%), limited capacity of local fiscal resources/medical insurance funds to support newly added items (50.0%), and difficulty standardizing distinctive TCM service items for project-approval (50.0%). From the management perspective, the most frequently suggested improvement was to refine differentiated project-approval standards for medical institutions at different levels (75.0%). Detailed results are summarized in Tables 15, 16.

**Table 15:** Key challenges in implementing the project guidelines for Jilin and Liaoning provinces.

Main difficulties in implementing the project-approval guidelines	Response		
	Number of cases	Percentage (%)	Percentage of cases (%)
Complex cost accounting for TCM services	17	25.8	70.8
Insufficient understanding of guideline requirements by medical institutions and non-standardized application materials	5	7.6	20.8
Difficulty standardizing distinctive TCM service items for project-approval	12	18.2	50.0
Conflicts in coordination with medical insurance payment policies	14	21.2	58.3
Coordination with subsequent fund supervision policies and definition of machine-review rules for TCM service items	6	9.1	25.0
Limited capacity of local fiscal resources/medical insurance funds to support newly added items	12	18.2	50.0
Total	66	100.0	275.0

**Table 16:** Areas requiring optimization in the project application guidelines for Jilin and Liaoning provinces.

Areas for simplifying the project-approval guidelines	Response		
	Number of cases	Percentage (%)	Percentage of cases (%)
Refine differentiated project-approval standards for medical institutions at different levels	18	35.3	75.0
Clarify pricing rules for TCM items and Western medicine items with the same name	13	25.5	54.2
Add a cost-accounting tool	11	21.6	45.8
Strengthen the trigger conditions for dynamic price adjustment	9	17.6	37.5
No need for simplification	0	0.0	0.0
Total	51	100.0	212.5

## 4 Discussion

### 4.1 Recognizing technical labor value within standardized pricing rules

The provider-side findings suggest that TCM pricing reform is closely related to perceived recognition of technical labor value. Professional title was negatively associated with pricing and payment satisfaction, while medical insurance payment restrictions were negatively associated with satisfaction and hospital management support was positively associated with satisfaction. These associations are consistent with value-based healthcare arguments that payment should reflect quality, outcomes, and the resources required to deliver care, rather than relying only on itemized inputs or material costs (5, 6). For TCM services, this issue is especially salient because clinical value is often embedded in syndrome differentiation, individualized treatment, manual techniques, treatment time, and practitioner experience. Standardized pricing item guidelines may improve administrative efficiency and facilitate insurance settlement, but they may also have difficulty capturing differences in procedures, technical complexity, schools of practice, and non-standardized diagnostic and therapeutic processes across TCM services (10). Therefore, the challenge is not simply whether prices should increase, but how pricing rules can recognize technical labor value while remaining administratively feasible and affordable for patients.

At the same time, these results should not be interpreted as evidence that current pricing rules directly caused provider dissatisfaction. The cross-sectional design can only identify associations. The negative association between guideline coverage and satisfaction may reflect providers’ greater exposure to the practical boundaries of standardized pricing items, or local difficulties in translating broad guidelines into institution specific services. Future studies should combine cost-accounting data, qualitative interviews, and longitudinal policy evaluation to clarify these mechanisms.

### 4.2 Technical barriers and resource allocation across institutional levels

The implementation barrier models had low explanatory power, but they still point to an important implementation issue: institutional context matters. Organizational level was associated with the barrier that traditional or ethnic medical techniques were not included in the project guidelines, and the direction of the findings suggests that institutions at different levels may experience pricing reform differently. The primary care reform and healthcare resource allocation depend not only on national policy design but also on local institutional capacity, workforce structure, and regional resource distribution (13–15).

These findings imply that uniform pricing item rules may require differentiated implementation support. As primary care institutions are important providers of chronic disease management and rehabilitation services, pricing mechanisms that do not adequately reflect their cost structures and service capacity may weaken provider incentives and limit the contribution of primary care to chronic disease management and rehabilitation services (16). Primary care institutions may therefore need training, technical guidance, referral support, and clearer service-scope standards before they can benefit from pricing reforms in the same way as tertiary hospitals. However, because the adjusted *R*^2^ values of the implementation barrier models were low, this conclusion should be treated as an equity-oriented interpretation rather than a strong predictive claim.

### 4.3 Patient affordability and varied stated pricing preferences

The patient-side findings show that only 0.8% of patients reported noticing changes in TCM service names or billing methods, indicating that most patients did not directly perceive the technical details of pricing reform. Their responses therefore reflect recent service experience, perceived effectiveness, price burden, and affordability rather than direct awareness of policy changes. Although 54.0% selected moderately lower prices, 39.5% supported moderate price increases if service quality improved and 37.9% supported tiered pricing. This pattern suggests that patients do not simply pursue lower prices but may seek value for money when higher prices are linked to visible improvements in service quality, price transparency, and insurance protection. However, the exploratory logistic models did not identify significant predictors of these stated preferences. This lays the groundwork for public acceptance of a differentiated pricing mechanism for TCM services in the future.

Furthermore, patients’ preferences regarding the pricing of TCM services vary and are influenced by factors such as demographics, the healthcare system, and service experiences (17, 18). Therefore, patient-side findings in this study should be interpreted as evidence of varied stated pricing preferences rather than estimates of actual monetary willingness to pay.

### 4.4 Coordination between pricing reform and insurance payment

The effectiveness of price reforms for TCM services is closely related to the coordination and alignment of medical insurance reimbursement policies (19). Regression results showed that medical insurance payment restrictions had strong negative association with healthcare professionals’ pricing and payment satisfaction, and administrators also identified conflicts between pricing reform and insurance payment as a major implementation difficulty. Health insurance payment and reimbursement arrangements can shape service delivery incentives, transparency, cost control, and access (4, 7, 8). Pricing reform may recognize the value of TCM services on paper, but if reimbursement caps, covered-session limits, or payment categories are not adjusted accordingly, part of the financial pressure may shift to patients or providers. In such cases, patients may face higher out-of-pocket costs due to limited reimbursement coverage and may be less able to benefit from the potential benefits of the reform. Meanwhile, provider incentives may be weakened if medical insurance payments do not adequately cover the costs associated with price adjustments.

This coordination issue is particularly relevant for TCM because some services involve individualized diagnosis, repeated treatment, rehabilitation, and long-term health management. These features may not always fit neatly into payment categories designed around standardized disease groups (20, 21). However, this study did not directly evaluate DRG payment design or insurance fund sustainability. Therefore, the findings should be interpreted as survey-based evidence for the need to coordinate pricing and payment reform, rather than as a direct assessment of any specific reimbursement model.

## 5 Suggestions

### 5.1 Develop evidence-informed value-based cost accounting

The findings suggest that TCM service pricing reform should move beyond purely item-based pricing and better recognize the technical labor value embedded in TCM services. Pricing authorities could further develop cost-accounting methods that capture TCM service characteristics, including labor time, technical complexity, occupational risk, service quality, and clinical outcomes (3, 11, 12). Such methods could help address some limitations of pricing approaches based mainly on material consumption or standardized service items by incorporating the physician’s technical input, labor costs, risk level, and service complexity as important pricing factors (5).

However, value-based pricing should be introduced cautiously through pilot evaluation and dynamic adjustment, with attention to both provider incentives and patient affordability. Within the framework of existing pricing item guidelines, policy pilots could explore differentiated pricing mechanisms (11). Rather than relying only on professional title, differentiated pricing mechanisms could consider service type, provider qualifications, quality standards, and efficacy evaluation, so that price adjustments are linked to demonstrable service value. This approach may help recognize complex TCM techniques more reasonably while avoiding excessive financial burden on patients. If carefully designed, this approach may help recognize the value of complex TCM techniques more reasonably, strengthen incentives for high-quality TCM clinical practice, and support the gradual development of a more evidence-informed and efficient TCM service pricing mechanism (6, 22).

### 5.2 Support safe differentiated access and pricing across institutional levels

Because institutional context was related to perceived implementation barriers, pricing reform should be accompanied by differentiated support for institutions at different levels (13–15). For primary care institutions, this may include targeted training, technical supervision from higher-level hospitals, clearer access standards for appropriate TCM techniques, and digital consultation support (23). Under the premise of clinical safety and quality control, policymakers could consider moderately relaxing equipment and professional title requirements for selected standardized and appropriate TCM techniques when they are provided as basic specialized services in primary care settings. At the same time, institutional-tier adjustment coefficients or other differentiated pricing arrangements could be explored in pilot settings to help compensate primary care institutions for higher unit operating costs caused by limited economies of scale. Such adjustments should be linked to defined service scopes, practitioner competency requirements, supervision mechanisms, and referral pathways, rather than to a general lowering of technical thresholds. Differentiated pricing or access rules should match service scope, institutional capacity, and quality control mechanisms so that primary care providers can participate in reform without compromising patient safety.

### 5.3 Strengthen patient value communication and service experience

Given that most patients did not directly notice changes in service names or billing methods but still expressed sensitivity to service quality and out-of-pocket burden, it is important to strengthen patient engagement and value communication. Because the technical value of healthcare services is often difficult for patients to quantify, medical institutions should improve communication with patients about the content, time input, technical difficulty, expected benefits, and out-of-pocket costs of TCM services. Transparent explanations may help patients understand why some services differ in price. However, communication alone is not enough. Medical institutions could systematically disclose information such as the technical difficulty classification of TCM services, procedure duration, human resource input, herbal medicine cost components, and expected clinical benefits in prominent locations within their facilities and on online platforms. This may help patients better understand the multidimensional basis for pricing and make the abstract value of technical services more understandable (18).

Improving service processes may also strengthen patients’ understanding of quality-linked price adjustments. TCM institutions could improve the patient experience by allowing sufficient communication time, explaining the rationale for syndrome-differentiated diagnosis and treatment, optimizing the treatment environment, and providing personalized health management guidance where appropriate. When patients can more clearly perceive the time, technical input, and professional expertise involved in TCM care, they may be more likely to understand tiered pricing or quality-linked price differences. Because patient stated preferences were varied and the exploratory models did not identify stable predictors, future tiered pricing policies should be combined with appropriate safeguards, such as clear price disclosure, insurance reimbursement coordination, and protection for patients with high treatment needs or limited ability to pay (17, 18).

### 5.4 Strengthen coordination between pricing reform and insurance payment policies

Because medical insurance payment restrictions showed a strong negative association with pricing and payment satisfaction, coordinated adjustment of medical insurance payment standards and healthcare service prices should be considered. When TCM service prices are revised, policymakers should assess the possible effects on insurance funds, provider behavior, patient out-of-pocket burden, and service accessibility (12). For TCM services commonly used in chronic disease management, rehabilitation, acupuncture, tuina, and other repeated treatment scenarios, reimbursement caps and covered-session limits may need periodic review. Payment models with TCM-specific clinical logic should be explored cautiously, supported by monitoring data and local pilot evidence, so that price reform does not simply transfer cost pressure from institutions to patients (20, 21, 24). Such coordination may help prevent the potential benefits of pricing reform from being offset by reimbursement restrictions and may better align provider incentives with patient affordability.

At the same time, medical institutions could strengthen communication and collaboration with regulatory authorities and participate in research on clinical pathways for TCM-specific disease groups. Such work may support the gradual development of payment categories that better reflect TCM diagnostic and treatment logic while meeting medical insurance management requirements and local realities (24, 25). This should be pursued through pilot studies and policy evaluation so that provider incentives and patient affordability can be balanced at the institutional level.

## 6 Conclusion

TCM service pricing reform is a health system coordination issue linking healthcare service prices, provider incentives, patient affordability, hospital management, and medical insurance payment. Based on survey evidence from Jilin and Liaoning provinces, this study found that professional title, insurance payment restrictions, hospital management support, and guideline coverage were associated with healthcare professionals’ pricing and payment satisfaction. Implementation barrier models suggested that institutional context and coverage of traditional or ethnic medical techniques may be relevant to perceived policy practice disconnects, although these models had limited explanatory power. Patient responses indicated limited direct awareness of pricing item changes but showed varied stated preferences regarding quality-linked price increases and tiered pricing. These findings suggest that future TCM pricing reform should strengthen value-based cost accounting, provide differentiated implementation support for institutions at different levels, enhance communication of service value to patients, and improve coordination between pricing and insurance payment policies. Because the data are cross-sectional and self-reported, these conclusions should be interpreted as exploratory evidence rather than causal claims.

### 6.1 Limitations

This study has several limitations. First, the cross-sectional, self-reported survey design supports the identification of associations but does not allow causal inference, and recall bias may have influenced responses. Second, the study was conducted only in Jilin and Liaoning provinces, which may limit generalizability to other regions of China or other health system settings. Third, the patient sample was modest and contained a high proportion of younger respondents, which may have introduced selection bias and limited more detailed willingness to pay modeling. Fourth, the questionnaire was designed for policy implementation assessment rather than as a fully validated psychometric instrument. Although items were discussed and revised by project-team members, no formal pilot testing or external expert review was conducted. Internal consistency was acceptable for the multi-item policy-familiarity and implementation barrier measures, but the main outcomes were single-item perception measures. Fifth, the healthcare professional questionnaire did not collect exact years of service, detailed department, income change, workload, or clinical specialty, leaving possible residual confounding from unmeasured factors. Sixth, patient-side pricing-preference results reflected stated preferences rather than actual payment behavior or monetary willingness to pay amounts. Finally, because several related outcomes and models were examined, multiple testing risk should be considered, and the low adjusted *R*^2^ values in several implementation barrier models indicate that important institutional, financial, and policy-context factors were not fully captured by the available variables.

## Data Availability

The raw data supporting the conclusions of this article will be made available by the authors, without undue reservation.
